# Comparing public and private hospitals in China: Evidence from Guangdong

**DOI:** 10.1186/1472-6963-10-76

**Published:** 2010-03-23

**Authors:** Karen Eggleston, Mingshan Lu, Congdong Li, Jian Wang, Zhe Yang, Jing Zhang, Hude Quan

**Affiliations:** 1 Shorenstein Asia-Pacific Research Center, Stanford University, Stanford, CA, USA; 2 Department of Economics, University of Calgary, Calgary, Alberta, Canada; 3 Jinan University Management School, Guangzhou, PR China; 4 Center for Health Management and Policy, Shandong University, Shandong, PR China; 5 Guangdong Bureau of Health Statistics Center, Guangzhou, PR China; 6 Department of Economics, University of Maryland, USA; 7 Department of Community Health Sciences and Centre for Health and Policy Studies, University of Calgary, Calgary, Alberta, Canada

## Abstract

**Background:**

The literature comparing private not-for-profit, for-profit, and government providers mostly relies on empirical evidence from high-income and established market economies. Studies from developing and transitional economies remain scarce, especially regarding patient case-mix and quality of care in public and private hospitals, even though countries such as China have expanded a mixed-ownership approach to service delivery. The purpose of this study is to compare the operations and performance of public and private hospitals in Guangdong Province, China, focusing on differences in patient case-mix and quality of care.

**Methods:**

We analyze survey data collected from 362 government-owned and private hospitals in Guangdong Province in 2005, combining mandatorily reported administrative data with a survey instrument designed for this study. We use univariate and multi-variate regression analyses to compare hospital characteristics and to identify factors associated with simple measures of structural quality and patient outcomes.

**Results:**

Compared to private hospitals, government hospitals have a higher average value of total assets, more pieces of expensive medical equipment, more employees, and more physicians (controlling for hospital beds, urban location, insurance network, and university affiliation). Government and for-profit private hospitals do not statistically differ in total staffing, although for-profits have proportionally more support staff and fewer medical professionals. Mortality rates for non-government non-profit and for-profit hospitals do not statistically differ from those of government hospitals of similar size, accreditation level, and patient mix.

**Conclusions:**

In combination with other evidence on health service delivery in China, our results suggest that changes in ownership type alone are unlikely to dramatically improve or harm overall quality. System incentives need to be designed to reward desired hospital performance and protect vulnerable patients, regardless of hospital ownership type.

## Background

The roles of the government and the private sector in health service delivery are controversial, especially in developing and transitional economies. Some authors argue for a dominant if not exclusive government role in health service delivery in developing countries [1]; others call for broad and expanding engagement with the private sector [2]. The relatively extensive literature comparing the performance of private not-for-profit, for-profit, and government providers mostly relies on empirical evidence from hospitals in high-income and established market economies [3-6]. Evidence from developing and transitional economies remains limited [7,8].

In China, although private ownership is common for outpatient services such as village clinics, private presence in inpatient delivery is limited and recent [9]. Policies begun in the late 1990s and reinforced by the 2009 health policy reforms [10]--which for the first time prominently called for expansion of private not-for-profit investments in health service delivery in China--set the stage for nationwide reform from almost universal government ownership of hospitals to more ownership types, albeit with government ownership still in the lead. Only a few studies have examined how public and private providers compete in China. Most patients may self-refer to a provider of their choice, and many aspects of operations appear similar across ownership types. For example, all Chinese clinics and hospitals employ physicians on their staff. Evidence suggests that providers respond to the incentives of the system in similar ways, such as avoiding unprofitable (public health) services and overproviding profitable high-tech diagnostic services and drugs [11-13]. Some studies find that private hospitals, seeking to attract patients in a public-dominated system of insurance and delivery, often charge prices that are lower than those of public hospitals, attract lower- and middle-income patients, and achieve higher patient satisfaction [13]. Similarly, Huang and colleagues [14] find that non-government hospitals charge prices that are generally lower than or equal to those of government hospitals. However, evidence specific to the quality of care provided in public versus private hospitals remains limited.

The aim of this study is to compare the operations and performance of public and private hospitals in Guangdong Province, China, focusing on differences in patient case-mix and quality of care. Such evidence can be valuable for understanding the role of the private sector in health service delivery in developing and transitional economies and for informing policy in countries that are contemplating how best to regulate mixed-ownership markets.

## Methods

### Sampling

We conducted a survey, approved and supported by the Division of Health Information of the Department of Health of Guangdong Province, to collect data on a sample of government-owned and private hospitals in Guangdong Province in 2004 and 2005. The data we collected contained only hospital-level aggregate information; there was no patient-level data. In addition, this project involved no intervention. According to the University of Calgary IRB definition, this qualifies for "not human subjects research" and does not requires an ethical approval. Hospitals participated in this survey granted consent to the Division of Health Information of the Department of Health of Guangdong Province to be included in this project.

Our sample was purposively constructed to oversample the private sector and cannot be considered a random sample of hospitals in that region. Specifically, the initial sampling design included all private for-profit hospitals officially registered by 2002 in five cities--Guangzhou, Zhongshan, Panyu, Jiangmen, and Dongguan--as well as a stratified random sample of not-for-profit hospitals in these same localities, stratified according to the official Chinese classification system for hospitals (*jibie*). The sample design called for random selection of one hospital out of five in the most selective category (level 3 first rank: *san ji jia deng*); one hospital out of 10 among lower-rank level-3 and level-2 categories; and one hospital out of 30 for the level-1 hospitals.

To take account of this sampling in the statistical analyses, we create indicator variables for hospital ownership categories (private not-for-profit, private for-profit, and government owned) and separate indicator variables for hospital levels of accreditation (*jibie *levels 1, 2, and 3) to use as explanatory variables in the multi-variate regression analyses (described further below). Since we do not claim that this sample is representative of the broader ownership mix and characteristics of hospitals in Guangdong Province or China as a whole, we do not weight the sample according to the stratified sampling design; to do so we believe would provide a false sense of accuracy, using what should be interpreted as a rich but limited sample of public and private hospitals in one region of China.

### Data collection

The data set includes data mandatorily reported by hospitals of all ownership types to the provincial health bureau, as well as information from an original survey instrument fielded in the fall of 2005 by enumerators who interviewed an administrator and collected administrative data by hand from each hospital. Our analytic sample includes 362 hospitals, both government and private, for which we have relatively complete data collected in 2005, reporting 2004 outcomes.

### Measurement of hospital characteristics

We use affiliation with a university as a proxy for teaching status. To designate a hospital as located in an urban or rural area, we used secondary data from Guangdong statistical yearbooks on the percentage of residents that are designated agricultural in each county or district [15,16]. Ninety-two hospitals, representing 25.4% of our sample, are located in areas where more than 50% of the population is agricultural; the remaining 270 are located in predominantly urban areas. We code whether each hospital is an "appointed hospital" for social insurance beneficiaries (*dingdian yiyuan*) based on secondary data from social insurance bureau documents.

### Data analysis

We compare hospitals using both univariate analyses and multi-variate regressions. Univariate analyses compare median and mean values across hospital categories (such as ownership type). Mean values are calculated as the average of individual hospital rates. To study whether differences among hospitals of different ownership types are statistically significant, we run regressions of the variable of interest on a categorical variable indicating ownership type (1, 2, 3) and report the *p *value of the ownership category coefficient in Table 1.

**Table 1:** Hospital characteristics*

	Government (N = 212)	Nongovernment nonprofit (N = 79)	Private for-profit (N = 71)	*P*-value
Rural (%)	27.8 (44.9)	24.0 (43.0)	19.7 (40.1)	0.1642
Acute-care (%)	83.0 (37.6)	62.0 (48.8)	71.8 (45.3)	0.0064
***Jibie *(Hospital accreditation level):**				0.0000
Not classified	32.4	75.0	87.3	
Level 1	21.9	5.3	9.9	
Level 2	32.4	18.4	2.8	
Level 3 (highest)	13.3	1.3	0.0	
Average number of beds	256.3 (308.4)	124.0 (155.9)	66.8 (114.0)	0.0000
Value of total hospital assets (RMB)	15,935.6 (29,091.3)	2,208.3 (3,080.0)	2,717.3 (11,831.5)	0.0000
Number of machines valued over 10,000 RMB	310.0 (611.7)	44.7 (63.5)	41.3 (70.7)	0.0000
**Staffing (averages):**				
Number of employees	355.1 (420.7)	120.1 (134.1)	89.0 (109.9)	0.0000
Number of physicians	114.1 (134.0)	32.8 (37.7)	24.0 (29.6)	0.0000
Number of nurses	115.8 (152.3)	32.9 (47.1)	24.8 (44.1)	0.0000
Number of pharmacists	23.6 (27.4)	7.3 (8.1)	4.8 (5.3)	0.0000
**Patient volume (averages):**				
Inpatient admissions	7,138.1 (8,395.0)	1,326.5 (3,081.4)	1,060.6 (3,139.7)	0.0000
Total outpatient visits	3,72,521.2 (4,19,051.5)	60,006.18 (83,287.9)	48,010.68 (1,12,454.4)	0.0000
Internal medicine	91,391.4 (1,29,503.7)	18,990.6 (31,165.4)	12,534.8 (28,439.2)	0.0000
Surgery	28,471.6 (41,400.0)	5,623.1 (8,904.8)	8,365.1 (18,076.1)	0.0000
Ob/Gyn	42,820.8 (51,862.3)	6,446.8 (11,973.2)	8,423.5 (23,649.6)	0.0000
Pediatrics	31,387.0 (79,364.0)	3,051.6 (6,022.9)	4,107.3 (11,671.7)	0.0000
Traditional Chinese medicine (TCM)	38,047.7 (68,017.8)	5,998.2 (9,839.4)	2,399.8 (4,128.2)	0.0000
Emergency room (ER) visits	42,532.5 (48,046.8)	7,711.5 (14,863.1)	6,158.5 (17,050.1)	0.0000
Average occupancy rate (%)	70.4 (33.5)	39.0 (32.9)	39.9 (35.0)	0.0000
Average length of stay (days)	22.0 (159.0)	61.2 (212.7)	14.6 (56.6)	0.8449
Average inpatient mortality rate (per 1000 admissions)	18.0248 (29.8192)	37.9922 (113.1023)	9.8640 (33.9575)	0.8337

***Note: ***The table reports variable means and standard deviations (in parentheses) for all variables except for *jibie *(hospital accreditation level), for which frequency is reported. The *p*-value column refers to the *p*-value on the coefficient of an ownership categorical variable (1, 2, 3) in a regression with the row variable as the dependent variable. A *p*-value of 0.0000 indicates that the *p*-value was less than 0.00005.

We further study the association between hospital ownership type and available measures of hospital structural and outcomes quality for the sub-sample of general-acute hospitals using the following multi-variate regression model:(1)

The dependent variable, *Y*, is a measure of each hospital's quality. Quality measures used in this study include structural and outcome measures and are all as continuous variables. Structural measures include four continuous variables: the value of a hospital's assets; the number of machines valued at over 10,000 RMB owned by a given hospital (measured by ln(number of machines valued over 10,000 RMB)); the total number of employees of the hospital (measured by ln(number of employees); and the number of physicians on a hospital's staff (measured by ln(number of physicians)). The outcome measure was one continuous variable: the inpatient mortality rate. The inpatient mortality rate is defined as the total number of patients who die during inpatient admission per 1,000 admissions to that hospital in that year. *N *and *F *are dummy variables indicating the hospital ownership type: *N *represents non-government non-profit ownership, and *F *indicates private for-profit ownership. (The omitted group is government ownership). *X *is a vector of hospital and market characteristics, such as *ln *(beds), indicators for whether the hospital is affiliated with a university or an appointed hospital for social insurance, and the percentage of the population that is agricultural in the hospital's county or district; and ξ is the error term. The coefficients *β*_1 _and *β*_2 _capture the effect on *Y *of non-government non-profit and for-profit ownership, respectively, relative to government ownership, controlling for other factors.

For some dependent and independent variables, logarithm transformation is used to normalize variable distribution. We take the natural logarithm of the inpatient mortality rate, for example, and use that converted form as the dependent variable for the econometric analysis to avoid an artificial bias in the statistical results from the skewed distribution of mortality rates. The importance of using such a transformation has been shown in previous analyses of hospital performance [17]. Ordinary least squares (OLS) regression is used in all the multi-variate regressions because all five dependent variables are continuous rather than categorical or count variables. All analyses were performed in Stata 9.0.

## Results

### Comparing hospital characteristics

As shown in Table 1, 212 (59%) of the hospitals in our sample are government-owned and 71 (20%) are private for-profit hospitals. Private hospitals, particularly for-profits, are less likely than their government counterparts to be general-acute hospitals and more likely to be specialty hospitals.

Compared to private hospitals, government hospitals are more concentrated in urban areas (*p *< 0.164), general acute care (p < .006), and tertiary services (level-3 *jibie *hospitals in the Chinese accreditation system) (*p *< 0.0005). Government hospitals are also much larger, in terms of beds, asset value, staffing, and outpatient and inpatient volume (*p *< 0.0005).

The average government hospital in our sample (*G*) has 256 beds and employs 114 physicians, compared to 124 beds and 33 physicians in the average non-government non-profit (*N*) and 67 beds and 24 physicians in the average private for-profit (*F*).

Even among general-acute hospitals, non-government hospitals are smaller: the median government hospital has 150 beds and 204 employees, including 74 physicians, whereas the median non-government non-profit hospital has 71 beds and 80 employees, and the median for-profit has 30 beds and 73.5 employees (data not shown). Government-owned and private for-profit hospitals have similar average numbers of employees per bed (1.3 and 1.4); non-government non-profit hospitals have fewer employees per bed, on average (120.1/124 = 0.97). Chinese hospitals hire about one pharmacist for every four or five physicians, regardless of ownership type.

Regarding level of accreditation, 57 hospitals are level-1 or community health centers; 84 are level-2; 29 are level-3, the highest level of tertiary care; and the remaining half (53%) of the sample are not classified. Non-classification may indicate that a hospital is too small or new to be prepared for accreditation review, did not have the staffing or equipment to qualify, or is waiting for results after applying for accreditation. Non-government hospitals are the majority of those at lower levels of accreditation or without a classification. The hospitals accredited at the top tertiary level (level 3) are virtually all government owned; only 1 (of 29) is a non-government non-profit.

Information about patient case-mix is only available for five broad categories of outpatient services. For outpatient surgical visits, private for-profit hospitals serve a higher proportion of patients than would be expected given their small size. The reverse is true for outpatient visits for traditional Chinese medicine, for which government hospitals provide the overwhelming majority of services.

The average occupancy rate is substantially higher at government hospitals (70%) than at non-government non-profits (39%) or for-profit hospitals (40%) (*p *< 0.000). Average length of stay is very heterogeneous (*p *< 0.845), with the highest level and standard deviation among non-government non-profit (and specialty) hospitals. Average reported mortality rates are highest for non-government non-profit hospitals and lowest for private for-profit hospitals, with government hospital mortality rates falling in between. Among general-acute hospitals, however, the average mortality rates for government and non-government non-profits do not statistically differ (Table 2).

**Table 2:** Inpatient mortality (number of deaths per 1,000 admissions) for general-acute hospitals (*N *= 307)

		Inpatient mortality central tendency		Inpatient mortality inter-quartile range	
Sub-groups of general-acute hospitals	N	Median	Mean	25th percentile	75th percentile
Ownership					
Government	176	9.5032	16.9060	5.3402	16.4919
Non-government non-profit	49	7.3910	19.0579	<0.0001	20.9790
Private for-profit	51	<0.0001	10.0783	<0.0001	6.5200
Size					
20--100 beds	115	4.0912	16.3998	<0.0001	9.4737
>100 beds	161	10.5263	15.7597	5.8560	16.2728
Contracted hospital for social insurance beneficiaries					
Not appointed	84	2.7911	9.7748	<0.0001	12.8986
Appointed (*dingdian*) hospital	192	8.6595	18.7615	4.5191	16.2873
Academic affiliation (proxy for teaching status)					
Non-university hospital	263	6.9565	15.7932	2.5100	14.3541
University hospital	13	17.4364	20.7440	12.8205	28.4837
Urban/rural location					
Urban	201	7.1259	18.6061	0.8300	17.0828
Rural	75	8.1411	9.1129	4.4577	13.0880
*Jibie *(Hospital accreditation level)					
Not classified	121	5.7358	16.3540	<0.0001	12.5829
Level 1 (community health center)	54	4.7862	10.5825	2.9674	8.6754
Level 2	76	10.8236	16.8193	6.2358	15.3246
Level 3 (highest tertiary care)	25	21.1464	23.7896	15.3653	28.7565

Over 80% of the government general-acute hospitals are appointed hospitals for social insurance beneficiaries, whereas the corresponding percentages for non-government non-profits and for-profits are 45% and 40%, respectively. Non-government hospitals are primarily established and controlled by social organizations, collectives, and firms. For-profit hospitals are much more likely than non-profits to be controlled by individuals.

To focus on comparing hospitals of differing ownership types that are otherwise quite similar, the remainder of the analyses restrict the sample to general-acute hospitals only (i.e., they exclude specialty hospitals).

### Comparing hospital case-mix and quality

Table 2 summarizes inpatient mortality rates (total number of patients who died during inpatient admission, per 1,000 admissions in the year 2004) across various categories of general-acute hospitals. These mortality rates reflect the combined effect of patient case-mix and quality of care.

Median inpatient mortality rates are higher in larger hospitals compared to smaller hospitals, insurance network ("appointed") hospitals compared to non-network hospitals, and university-affiliated hospitals compared to non-teaching hospitals. Median inpatient mortality rates increase with the official rank of the hospital, so that the highest-accredited (level-3) hospitals have the highest inpatient mortality rates.

### Multi-variate analysis of the correlates of hospital quality

Table 3 reports results of multi-variate regression analysis of the correlates of hospital structural quality as proxied by assets and staffing. In a regression exploring the correlates of the value of a hospital's total assets (measured with a log-transformed continuous variable), a 10% increase in beds was correlated with a 10.7% increase in assets. Controlling for this effect, government hospitals had a higher total value of assets than private hospitals. Being an appointed hospital in the social insurance network was associated with more assets. Table 3 next reports OLS regressions of hospital staffing, measured as continuous variables by taking the natural logarithms of the total number of employees (model 3) and of the number of physicians (model 4). Non-government non-profit ownership was associated with fewer total employees and fewer physicians, controlling for beds and social insurance appointment status (which were both associated with more personnel). For-profit private and government hospitals did not statistically differ in total employment, although for-profits had fewer medical professionals and more support staff.

**Table 3:** Correlates of structural quality (hospital assets and staffing), general-acute hospitals

	Model 1	Model 2	Model 3	Model 4
Dependent variable:	Assets (ln (value of assets in 10,000 RMB))	Number of machines valued over 10,000 RMB (ln (#machines valued over 10,000 RMB))	Total employees (ln (#employees))	Physicians (ln (#physicians))
Explanatory variables:				
Non-government non-profit (indicator variable)	-0.974 (6.69)**	-0.662 (3.87)**	-0.392 (4.20)**	-0.629 (5.70)**
Private for-profit (indicator variable)	-0.433 (2.62)**	0.024 (0.12)	-0.147 (1.37)	-0.329 (2.60)**
*ln *(beds)	1.073	0.886	0.729	0.657
	(19.19)**	(13.57)**	(20.09)**	(15.34)**
Contract with social insurance ("appointed" indicator variable)	0.513 (4.02)**	0.401 (2.67)**	0.245 (2.96)**	0.296 (3.03)**
University hospital (indicator variable)	0.1 (0.37)	0.409 (1.33)	0.148 (0.85)	0.259 (1.26)
Rural hospital (indicator variable)	0.002	0.189	0.016	-0.054
	(0.01)	(0.84)	(0.13)	(0.37)
Constant	3.022	-0.042	1.61	0.825
	(10.25)**	(0.12)	(8.42)**	(3.66)**
Observations	286	275	288	288
*R*-squared	0.74	0.58	0.73	0.66

***Note: ***Absolute value of *t *statistics in parentheses

* significant at 5%; ** significant at 1%

Each column presents a separate ordinary least squares (OLS) regression, with the dependent variable as listed in the column heading and the explanatory variables as listed in the first column; each cell of the table reports the estimated regression coefficient, with the absolute value of the associated t statistic in parentheses below the estimated coefficient. We take the natural logarithm of each dependent variable e.g., *ln *(value of assets in 10,000 RMB) and use that converted form for the econometric analysis to avoid an artificial bias in the statistical results from the skewed distribution of the continuous dependent variables.

Table 4 presents our OLS regression results for the log transformation of inpatient mortality rates. Case-mix controls include the number of emergency patients, the percentage of outpatient visits for five categories of outpatient service, the percentage of inpatient beds across several different departments, and the official accreditation level of the hospital. As for the other regressions, we also control for possible confounding from differences in the total number of hospital beds, urban or rural locations, inclusion in the social insurance network, and university affiliation. We find that mortality rates for non-government non-profit and for-profit hospitals did not statistically differ from those of government hospitals of similar size and patient mix. Factors significantly associated with higher mortality rates include being an appointed hospital for social insurance beneficiaries, having larger percentages of outpatient visits for internal medicine and traditional Chinese medicine, and having a higher percentage of inpatient beds in departments of internal medicine and tumors. Factors significantly correlated with lower inpatient mortality rates include having a higher percentage of beds devoted to surgery, obstetrics, and gynecology.

**Table 4:** Correlates of outcome quality with limited control for case-mix, general-acute hospitals

	Inpatient mortality rate	
	Model 1	Model 2
Non-government non-profit (indicator variable)	0.003 (0.48)	-0.005 (0.9)
Private for-profit hospital (indicator variable)	-0.012	-0.002
	(1.92)	(0.34)
*ln *(beds)	0.003	-0.001
	(1.3)	(0.34)
*ln *(emergency patients)	-0.005	-0.001
	(3.67)**	(1.2)
Contract with social insurance ("appointed" indicator variable)	0.012 (2.50)*	0.014 (3.33)**
University hospital (indicator variable)	-0.006	-0.012
	(0.64)	(1.31)
Rural hospital (indicator variable)	-0.004	-0.002
	(0.49)	(0.33)
Internal medicine visits as % of op visits		0.02
		(2.03)*
Surgery visits as % of op visits		-0.028
		(1.55)
Ob/gyn visits as % of op visits		0.011
		(0.42)
Pediatrics visits as % of op visits		0.041
		(1.33)
TCM visits as % of op visits		0.044
		(3.58)**
Internal medicine ip beds as % of beds		0.054
		(5.36)**
Surgery ip beds as % of beds		-0.027
		(2.40)*
Pediatrics ip beds as % of beds		-0.05
		(1.4)
Obgyn ip beds as % of beds		-0.045
		(2.49)*
Psychiatry dept ip beds as % of beds		-0.013
		(0.34)
Infectious disease dept ip beds as % of beds		-0.041
		(0.83)
Tumors ip beds as % of beds		0.292
		(4.11)**
TCM ip beds as % of beds		-0.007
		(0.92)
Hospital accreditation level 2 (indicator variable)		0.006 (1.04)
Hospital accreditation level 3 (highest; indicator variable)		0.002 (0.21)
Not classified into a level (indicator variable)		0.004
		(0.79)
Constant	0.044	0.019
	(3.54)**	(1.24)
Observations	265	265
R-squared	0.12	0.44

***Note: ***Absolute value of t statistics in parentheses

All dependent variables are analyzed in *ln *(.) form

op = outpatient; ip = inpatient

* significant at 5%; ** significant at 1%

Model 1 and Model 2 are separate OLS regressions, with the explanatory variables as listed in the first column; each cell of the table reports the estimated regression coefficient, with the absolute value of the associated t statistic in parentheses below the estimated coefficient. A blank cell for Model 1 indicates the row variables (e.g., "Internal medicine visits as % of op visits") that were not included in the Model 1 regression. We take the natural logarithm of the dependent variable (e.g., *ln *(inpatient mortality rate)) and use that converted form for the econometric analysis to avoid an artificial bias in the statistical results from the skewed distribution of mortality rates.

### Multi-variate analysis of other hospital characteristics

For completeness we mention additional statistical analyses that are not the primary focus of this article. Larger size, being an appointed hospital, and government control are all associated with higher volumes of both inpatient admissions and outpatient visits. Private ownership (regardless of profit status) is correlated with fewer outpatient visits overall for most sub-categories of visits. However, for outpatient *surgical *visits, for-profit hospital volume is not statistically different from that of government hospitals. Government control and appointed status are associated with more patients per doctor, more patients per nurse, and higher occupancy rates. Higher cooperative medical system coverage rates are associated with higher occupancy rates, suggesting that expanding insurance coverage to China's rural majority increases utilization of inpatient resources. All results and their associated statistical tests are available in the working paper [18].

## Discussion

In this study, we examined the overall operations of over 360 public and private hospitals in Guangdong Province in southern China. As one of the wealthier provinces with vibrant non-state firms in other sectors of its economy, Guangdong makes an interesting case study and may exemplify the path that other parts of China might take in the future.

We find that government-owned hospitals continue to dominate the highest levels of tertiary hospital care and remain the primary provider of outpatient traditional Chinese medicine services. Private hospitals are more likely to be specialty hospitals, and private for-profit hospitals aggressively cater to the surgical outpatient market, attracting a clientele in that area disproportionate to their size. Among general-acute hospitals, private hospitals appear to be scaled-down versions of their government-owned counterparts along most dimensions, from staffing and equipment to patient volumes and outcomes.

Consistent with previous literature [19,20], we find that hospitals have responded to the incentives implicit in China's fee-for-service payment system. For example, across all ownership forms, hospitals hire about one pharmacist for every four or five physicians (Table 1). Hospitals in China earn 40 to 50 percent of their revenues from drug sales [21], and the 2009 reform plan explicitly calls for reducing this reliance on pharmaceutical sales revenue.

Does the quality of care differ across ownership types? Quality of health care is fundamentally multi-dimensional and has long been difficult to measure. Our data only include measures of quality based on structural features (such as equipment and staffing) and some patient outcomes such as inpatient mortality rates, which are known to have limitations [22-25].

For structural quality, we find that compared with private general-acute hospitals (and controlling for hospital beds, urban location, insurance network, and university affiliation), government hospitals own more total assets and more pieces of expensive medical equipment and hire more employees, including physicians. Other factors associated with higher structural quality include having more beds and being an appointed hospital in the social insurance network. Government and for-profit private hospitals do not statistically differ in total staffing, although for-profits have proportionally more support staff and fewer medical professionals.

In univariate analysis, median inpatient mortality rates increase with the official rank (*jibie*) of the hospital. Since the level-3 hospitals are the most sophisticated and the largest tertiary hospitals, while level-1 and level-2 hospitals are of more modest scope and clinical reputation, that mortality rates increase with accreditation level clearly indicates that mortality rates are a measure of illness severity and patient case-mix as much as quality of care.

In our multi-variate analysis, case-mix controls include the number of emergency patients, the percentage of outpatient visits for five categories of outpatient services, the percentage of inpatient beds across several different departments, and the official accreditation level of the hospital. These hospital-level indicators remain imperfect because they reflect hospital management decisions as well as case-mix, and they do not capture differences in severity of disease within service categories. Nevertheless, these controls help to disentangle the effect of ownership type from that associated with serving different patient clienteles. For example, it seems perfectly plausible that hospitals that treat cancer patients have higher mortality rates than those that specialize in childbirth and/or routine surgeries, as our results indicate (Table 4).

With these admittedly limited controls for case-mix, inpatient mortality rates at non-government non-profit and for-profit hospitals do not statistically differ from government hospitals of similar size and patient mix. Thus we conclude that there is no evidence in our sample that non-government hospitals are of systematically better or worse quality than Chinese government hospitals.

These results contribute to the literature on whether public and private hospitals in developing and transitional economies differ in quality of care. Although economic theories provide a valuable conceptual framework for how ownership affects quality, they do not provide a single prediction (see [5] for a review). For example, one prominent theory of ownership [26] predicts that private providers will invest more in cost control but may be of either higher or lower quality than their government-owned counterparts.

How quality differs with ownership is therefore an empirical question. But the empirical evidence from high-income and established market economies is mixed [4,5], and the literature on private providers in developing and transitional economies [7,8,28-30] rarely studies quality of care. As Mills and colleagues [8] point out, policymakers frequently have concerns about the uneven quality of private outpatient providers. The scope of the private sector in inpatient care services is often more limited. Palmer and Mills [31] highlight the importance of contractual relationships. A recent systematic review [32] finds a positive impact of private providers on the quality of primary or public health services for the poor. Loevensohn and Harding [2] argue that contracting for health service delivery from non-government organizations can improve quality. Yet the success stories they emphasize all focus on primary care, not inpatient services.

One of the few studies that examine public and private delivery of hospital services [33] finds that contracting out management of three rural district hospitals to private for-profit management in South Africa was associated with higher nursing quality but lower structural quality and no difference in maternal and perinatal mortality rates compared to three paired public hospitals. Our study provides a larger sample size for comparing quality in public and private hospitals from the largest developing and transitional economy.

In China government-owned hospitals continue to dominate the "commanding heights" of inpatient health service delivery, in contrast to the mixed ownership markets of some developing and many higher-income countries, where government hospitals play a more dominant role in safety net and specialty services and in rural areas. Although government hospitals in our Guangdong sample have higher measures of structural quality along most dimensions compared to non-government hospitals, multi-variate analyses suggest that outcome quality does not systematically differ by ownership type after taking account of the different case-mix of patients seeking care in different hospitals.

### Study Limitations

Although we analyze one of the most detailed data sets available to date on government and non-government hospitals in China, data limitations are numerous. The analysis and conclusions must be interpreted with caution in light of the deliberate oversampling of urban and periurban areas and private for-profit hospitals, and the quasi-random sample design. The data cannot be considered representative of Guangdong Province or the country as a whole.

Even information on ownership or location can be subject to error. For example, we use the administrative locality of a hospital's address to classify hospitals as urban or rural, but this measure is far from synonymous with a hospital's true market. Another example is ownership categorization. The category "non-government non-profit" should be interpreted with caution; these hospitals represent a heterogeneous mixture of non-governmental organizations and providers that may be controlled by a government agency but operated as a semi-autonomous unit.

We analyze imperfect proxies for hospital structure and outcome quality. The available information does not include patient-level data, hospital-level measures of patients' insured or uninsured status, disease severity, or number of admissions by disease category (although we do know how outpatient visits are distributed across five broad areas of service and whether the hospital is appointed within the social insurance network). Hospitals with a more severe case-mix will have higher mortality rates, even if they are providing exemplary quality of care. More detailed analysis, using data at the patient level before and after a plausibly exogenous policy change, would be of value to determine the causal effect of ownership on quality, rather than the correlations observed in cross-sectional data.

## Conclusion

Comparing hospital operations across multiple dimensions for over 360 public and private hospitals in Guangdong Province, we find that private hospitals are generally smaller and attract a less severe case-mix, in part because they are newer market entrants and less likely to be in the network of providers contracted by social health insurance programs. Mortality rates are higher in larger, more highly accredited, and government-owned hospitals that treat a more severe case-mix of patients. In descriptive multi-variate regression analyses, controlling for hospital characteristics and available measures of case-mix, mortality rates do not statistically differ between government and private hospitals.

In combination with other evidence on health service delivery in China [10-14,20], we conclude that ownership reforms alone are unlikely to dramatically improve or harm quality. System incentives need to be designed to reward desired hospital performance and protect vulnerable patients, regardless of hospital ownership type.

The future of mixed-ownership delivery in China remains uncertain. The plan for reform of China's health system announced in April 2009 [10] calls for continued dominance by public providers over most service delivery, while simultaneously calling for increased non-government investment in both financing and delivery. As reforms unfold, the challenge will be to harness the potential innovation, efficiency, and responsiveness of private providers, while enhancing capacity to regulate and monitor in order to assure equitable access and avoid unhealthy market segmentation. China's response to this challenge in the next few years will shape the equity and efficiency of the health-care system for decades to come.

## Competing interests

The authors declare that they have no competing interests.

## Authors' contributions

KE participated in the study design, survey instrument construction, data collection and analysis, and wrote the first draft of the manuscript. ML contributed to the study design, statistical analysis, and interpretation, as well as writing of the manuscript. CL, JW, and ZY contributed to the survey and data collection. JZ contributed to the data management and analysis. HQ contributed to the interpretation and writing of the manuscript. All authors read and approved the final manuscript.

## Pre-publication history

The pre-publication history for this paper can be accessed here:

http://www.biomedcentral.com/1472-6963/10/76/prepub
